# Supplementary material for: Factors Determining Nestedness in Complex Networks
Source: PLoS One. 2013 Sep 19;8(9):e74025. doi: 10.1371/journal.pone.0074025 (PMC3777946; doi:10.1371/journal.pone.0074025)
Supplement: Appendix S1 — In this appendix we show how to generalize the new nestedness index to bipartite networks. (PDF) [file pone.0074025.s001.pdf]

Appendix S1: Nestedness index for bipartite networks

Mutualistic networks are usually bipartite: two sets of nodes exist such that all edges are between nodes in one set and those of another. The ones considered in Ref. [1], for instance, are composed of animals and plants which interact in symbiotic relations of feeding-pollination; these interactions only take place between animals and plants. Let us therefore consider a bipartite network and call the sets Γ_1 and Γ_2, with n_1 and n_2 nodes, respectively ($n_1 + n_2 = N$). Using the notation $\langle\cdot\rangle_i$ for averages over set Γ_i, the total number of edges is $\langle k\rangle_1 n_2 = \langle k\rangle_2 n_1 = \frac{1}{2}\langle k\rangle N$. Assuming that the network is defined by the configuration ensemble, though with the additional constraint of being bipartite, the probability of node l being connected to node i is

$$\hat{\epsilon}_{il} = 2\frac{k_i k_l}{\langle k\rangle N}$$

if they belong to different sets, and zero if they are in the same one. Proceeding as before, we find that the expected value of the nestedness for a bipartite network is

$$\eta_{bip} = \frac{1}{N^2}\left[\sum_{i,j\in\Gamma_1}\frac{1}{k_i k_j}\sum_{l\in\Gamma_2}\frac{k_i k_l}{\langle k\rangle_1 n_2}\frac{k_l k_j}{\langle k\rangle_2 n_1} + \sum_{i,j\in\Gamma_2}\frac{1}{k_i k_j}\sum_{l\in\Gamma_1}\frac{k_i k_l}{\langle k\rangle_1 n_2}\frac{k_l k_j}{\langle k\rangle_2 n_1}\right] = \frac{n_1\langle k^2\rangle_2 + n_2\langle k^2\rangle_1}{\langle k\rangle_1\langle k\rangle_2(n_1+n_2)^2}. \quad (1)$$

Interestingly, if $n_1 = n_2$, the fact that the network is bipartite has no effect on the nestedness: $\eta_{bip} = \eta_{conf}$.

References

1. Bastolla U, Fortuna M, Pascual-García A, Ferrera A, Luque B, et al. (2009) The architecture of mutualistic networks minimizes competition and increases biodiversity. Nature 458: 1018-21.
