# Supplementary material for: Factors Determining Nestedness in Complex Networks
Source: PLoS One. 2013 Sep 19;8(9):e74025. doi: 10.1371/journal.pone.0074025 (PMC3777946; doi:10.1371/journal.pone.0074025)
Supplement: Appendix S2 — This appendix explains how the Pearson’s correlation coefficient scales with size in finite scale-free networks. (PDF) [file pone.0074025.s002.pdf]

Appendix S2: Finite Size Scaling of Pearson's coefficient in scale-free networks

As show in Fig.2, finite-size networks built with the configuration model have a non-vanishing value of Pearson's coefficient, r. Given the fact that these networks are constructed without assuming any type of correlations, this is necessarily a finite-size effect. Let us compute r explicitly in finite-size scale-free networks, with $P(k) \propto k^{-\gamma}$ with $2 < \gamma < 3$. The maximum expected degree, k in a network of size N is of the order $N^{\frac{1}{\gamma-1}}$ and this cut-off controls the scaling of moments $< k^m > \sim \int_1^{K_{max}} k^m k^{-\gamma} dk \sim k^{m-\gamma+1}|_1^{N^{\frac{1}{\gamma-1}}} \sim N^{\frac{m-\gamma+1}{\gamma-1}} - 1$. Combining the expressions for the first three moments appearing in the definition of r, Eq.7, one readily obtains;

$$r = \frac{aN^{\frac{3-\gamma}{\gamma-1}} - bN^{2\frac{3-\gamma}{\gamma-1}}}{cN^{\frac{4-\gamma}{\gamma-1}} - dN^{2\frac{3-\gamma}{\gamma-1}}} \sim -eN^{\frac{2-\gamma}{\gamma-1}} \qquad (1)$$

where a, b, c, d, and e are un-specified positive constants. For a scale-free network with $\gamma = 2.5$ this reduces to $r \sim N^{-\frac{1}{3}}$ in agreement with numerical results shown in Fig.2 (observe that, as we use a logarithmic scale, the absolute value of r rather than r itself is employed).
