# Supplementary material for: Factors Determining Nestedness in Complex Networks
Source: PLoS One. 2013 Sep 19;8(9):e74025. doi: 10.1371/journal.pone.0074025 (PMC3777946; doi:10.1371/journal.pone.0074025)
Supplement: Appendix S3 — This appendix illustrates how to sample networks with a given value of the Pearson’s correlation coefficient. (PDF) [file pone.0074025.s003.pdf]

Appendix S3: Sampling networks with a given value of r

Given that networks with very large r (in absolute value) are rare, and thus they seldom appear in the randomization process used to built the configurational ensemble (or null model) we have implemented the Wang-Landau (multi-canonical) algorithm to enrich the sampling with such rare networks [1]. The gist of this technique is to perform a "random" walk in the r-space, in such a way that jumps toward frequently visited r-values are penalized and, instead, rarely visited r's are favoured, which requires storing the statistics of the number of times every value of r has been previously "extracted". Starting from an initial network (with r_1), a small change in its topology is tentatively made, and the resulting new network (with r_2) is accepted with probability

$$P(r_1 \to r_2) = min\left[\frac{g(r_1)}{g(r_2)}, 1\right] \tag{1}$$

where $g(r)$ stands for the (previously observed) frequency. This algorithm allows for uniform searches in "r-space".

For the three different types of degree-distributions considered above, we proceed as follows:

- Generate a network ($N = 50$) with the configuration model with a fixed degree sequence.
- Explore the r-space using the Wang-Landau algorithm (up to, at least, 4000 different visits to the most frequent states).
- Average over 500 independent realizations.

While running the search in the r-space, we also measure and store the nestedness values of Eq.(6) for every (binned) value of r; after properly normalizing, we obtain the averaged value of nestedness as a function of r as shown in Fig.2 .

In all three cases, we obtain a very clear (almost linear) dependence between r and η: disassortative networks are distinctly nested (on average) while assortative ones are anti-nested (on average). Let us caution that this conclusion holds "on average", i.e. our results do not necessarily imply that any particular disassortative network is actually nested.

For the case of scale free networks we have been able to analytically determine the relation between the averaged Pearson's coefficient and the averaged nestedness. Results are illustrated in Figure S1, which shows the value of $\overline{\eta}_\beta$ (given by Eq. (6) in Appendix S3) against the assortativity r for various scale-free networks. Nestedness is seen to grow very fast with increasing disassortativity (decreasing negative r), while in general slightly assortative networks are less nested than neutral (uncorrelated) ones, i.e. they are "anti-nested". Note, however, that highly heterogeneous networks (scale-free with $\gamma \to 2$) show an increase in $\overline{\eta}_\beta$ for large positive r. Analogously to what was done for the configuration model, we compute the average nestedness in this second ensemble for a fixed value of r. In particular, we have considered the same 16 workbench networks as above, and produced Figure S 1, where we show for each network, the averaged nestedness (with its corresponding standard deviation) as a function of r. The empirical values of r, η are marked with black crosses.

In this new, more constrained ensemble the null model performs only slightly better than the configurational one: 63% within one standard deviation (as opposed to 42% in the first null model), 85% within two (versus 72%), and 92% for three standard deviations (as opposed to 82%). Observe that, also here, empirical values are reasonably well explained by randomized values, in almost all cases.

References

1. Wang F, Landau D (2001) Efficient multiple-range random walk algorithm to calculate the density of states. Physical Review Letters 86: 2050-2053.

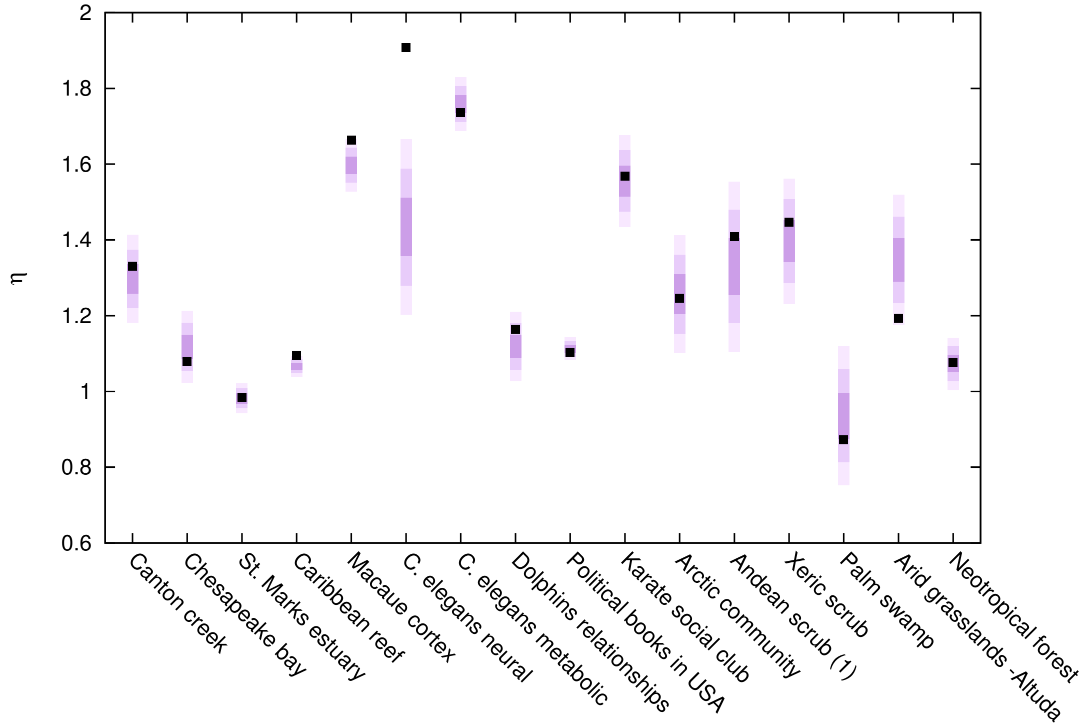

Figure S 1. Averaged nestedness index, η, as measured in in the second null model in which the value of r is preserved. The actual value of η in the real network is marked with a black square, while the coloured intervals corresponds to one, two and three standard deviations respectively. In most cases the empirical value lies in or near the corresponding interval. Allowing for two or three standard deviations essentially all empirical points yield within the corresponding interval.
