# Supplementary material for: Factors Determining Nestedness in Complex Networks
Source: PLoS One. 2013 Sep 19;8(9):e74025. doi: 10.1371/journal.pone.0074025 (PMC3777946; doi:10.1371/journal.pone.0074025)
Supplement: Appendix S4 — In this appendix we analytically compute degree-degree correlations in heterogeneous networks. (PDF) [file pone.0074025.s004.pdf]

Appendix S4: Degree-degree correlations in heterogeneous networks

It has been recently shown [1] that there is a mapping between any mean-nearest-neighbour function $\overline{k_{nn}}(k)$ –accounting for degree-degree correlations– and its corresponding mean-adjacency-matrix $\hat{\epsilon}$, which is as follows:

$$\overline{k_{nn}}(k) = \frac{\langle k^2 \rangle}{\langle k \rangle} + \int d\nu f(\nu) \sigma_{\nu+1} \left[\frac{k^{\nu-1}}{\langle k^\nu \rangle} - \frac{1}{k} \right]. \tag{1}$$

This can be seen as an expansion of $\overline{k_{nn}}(k)$ in powers of k with some weight function f and $\sigma_{\nu+1} \equiv \langle k^{\nu+1} \rangle - \langle k \rangle \langle k^\nu \rangle$ (which can always be done [1]), the corresponding matrix $\hat{\epsilon}$ takes the form

$$\hat{\epsilon}_{ij} = \frac{k_i k_j}{\langle k \rangle N} + \int d\nu \frac{f(\nu)}{N} \left[\frac{(k_i k_j)^\nu}{\langle k^\nu \rangle} - k_i^\nu - k_j^\nu + \langle k^\nu \rangle \right]. \tag{2}$$

Without entering here the details of this decomposition (for which we refer the reader to Ref. [1]) let us just remark that the first term in Eq.(2) coincides with the expected value for the standard configuration model, while the second one accounts for correlations. Hence, Eq.(2) can be seen as an extension of the configuration model including correlations, i.e. a *correlated configuration model.* In particular, Eq.(2) encodes the way a network should be wired (i.e. the probabilities with which any pair of nodes should be connected) to have the desired degree sequence and degree-degree correlations.

In many empirical scale-free networks, $\overline{k_{nn}}(k)$ can be fitted by $\overline{k_{nn}}(k) = A + Bk^\beta$, with $A, B > 0$ [2–4] – the mixing being assortative (disassortative) if β is positive (negative). Such a case is described by Eq. (1) with $f(\nu) = C[\delta(\nu - \beta - 1)\sigma_2/\sigma_{\beta+2} - \delta(\nu - 1)]$, with C a positive constant, which simplifies significantly the expressions above. This choice yields

$$\overline{k_{nn}}(k) = \frac{\langle k^2 \rangle}{\langle k \rangle} + C\sigma_2 \left[\frac{k^\beta}{\langle k^{\beta+1} \rangle} - \frac{1}{\langle k \rangle} \right] \tag{3}$$

After plugging Eq. (3) into Eq. (7) in the main text, one obtains:

$$r = \frac{C\sigma_2}{\langle k^{\beta+1} \rangle} \left(\frac{\langle k \rangle \langle k^{\beta+2} \rangle - \langle k^2 \rangle \langle k^{\beta+1} \rangle}{\langle k \rangle \langle k^3 \rangle - \langle k^2 \rangle^2} \right). \tag{4}$$

It turns out that the configurations most likely to arise naturally (i.e those with maximal entropy) usually have $C \simeq 1$ [1]. Therefore, and for the sake of analytical simplicity, we shall consider this particular case (note that $C = 1$ corresponds to removing the linear term, proportional to $k_i k_j$, in Eq. (2), and leaving the leading non-linearity, $(k_i k_j)^{\beta+1}$, as the dominant one); that is, we shall use

$$\hat{\epsilon}_{ij} = \frac{1}{N} \left\{ \frac{\sigma_2}{\sigma_{\beta+2}} \left[\frac{(k_i k_i)^{\beta+1}}{\langle k^{\beta+1} \rangle} - k_i^{\beta+1} - k_j^{\beta+1} + \langle k^{\beta+1} \rangle \right] + k_i + k_j - \langle k \rangle \right\}. \tag{5}$$

Substituting the adjacency matrix for this expression in the definition of η (Eq.(6) in the main text), we obtain its expected value as a function of the remaining parameter β:

$$\overline{\eta}(\beta) = \frac{\langle k \rangle^2}{\langle k^2 \rangle} \left[1 + (\sigma_2 - \alpha_\beta^2 \rho_\beta) \left(2 \frac{\langle k^\beta \rangle \langle k^{-1} \rangle}{\langle k^{\beta+1} \rangle} - \langle k^{-1} \rangle^2 \right) + \alpha_\beta^2 \rho_\beta \left(\frac{\langle k^\beta \rangle}{\langle k^{\beta+1} \rangle} \right)^2 \right], \tag{6}$$

where $\alpha_\beta \equiv \sigma_2/\sigma_{\beta+2}$ and $\rho_\beta \equiv \langle k^{2(\beta+1)}\rangle - \langle k\rangle^{2(\beta+1)}$. Note that $\overline{\eta}_0 = 1$, as corresponds to uncorrelated networks. As r can be inferred from β using Eq.(4), then we can plot the resulting η as a function of r for different networks. In particular, for scale-free networks with $P(k) \sim k^{-gamma}$ we obtain the curves shown in Fig.S1; they exhibit a clear tendency (at least for $\gamma > 2$): disassortative networks tend to be nested and the or the way around.

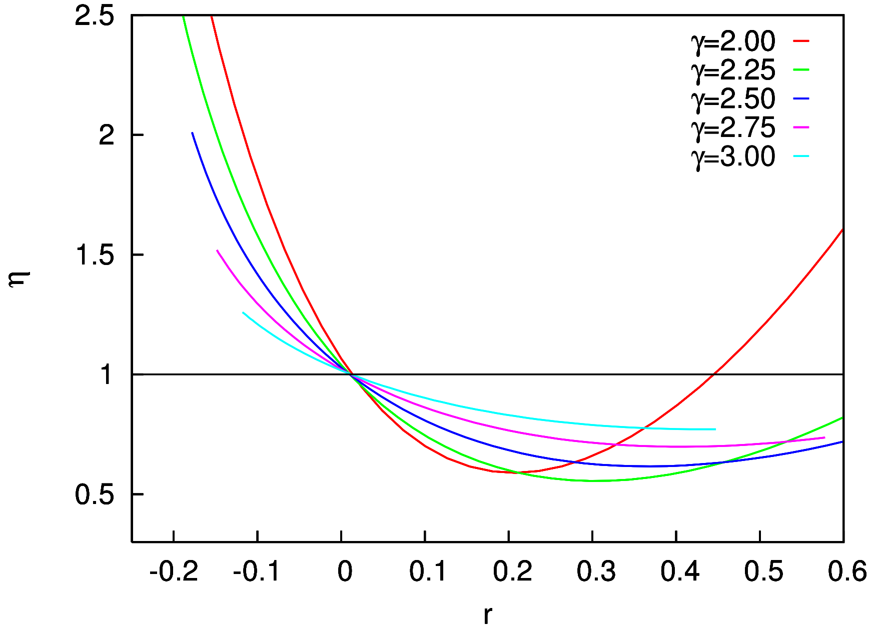

Figure S2. Nestedness against assortativity (as measured by Pearson's correlation coefficient, r) for scale-free networks with different values of the degree-distribution exponent, γ. $\langle k\rangle = 10$, $N = 1000$.

References

1. Johnson S, Torres J, Marro J, Muñoz MA (2010) Entropic origin of disassortativity in complex networks. Phys Rev Lett 104: 108702.

2. Capocci A, Caldarelli G, de los Rios P (2003) Quantitative description and modeling of real networks. Phys Rev E 68: 047101.

3. Boccaletti S, Latora V, Moreno Y, Chavez M, Hwang D (2006) Complex networks: Structure and dynamics. Phys Rep 424: 175.

4. Pastor-Satorras R, Vázquez A, Vespignani A (2001) Dynamical and correlation properties of the internet. Phys Rev Lett 87: 258701.
