# Supplementary material for: Factors Determining Nestedness in Complex Networks
Source: PLoS One. 2013 Sep 19;8(9):e74025. doi: 10.1371/journal.pone.0074025 (PMC3777946; doi:10.1371/journal.pone.0074025)
Supplement: Appendix S5 — This appendix contains tables with the network data used in the manuscript. (PDF) [file pone.0074025.s005.pdf]

Appendix S5: Network Data

Unimodal networks on Fig 4

Network	r	r z-score	r P-value	η	η z-score	η P-value	η z-score with correlations
Biological - Food Webs (13)							
Little Rock lake	-0.327	0.514 ()	0.300 ()	1.326	0.026 ()	0.199 ()	0.350 ()
Ythan Estuary	-0.278	2.000 ()	0.039 (*)	1.482	-2.459 ()	0.021 (*)	-1.339 ()
Stony Stream	-0.208	0.368 ()	0.401 ()	1.295	-0.500 ()	0.300 ()	-0.594 ()
Canton creek	-0.201	0.667 ()	0.266 ()	1.331	-1.454 ()	0.096 ()	-0.880 ()
Skipwith Pond	-0.086	-3.406 (*)	0.001 (*)	1.014	2.142 ()	0.017 (*)	-4.600 (*)
El Verde rainforest	-0.171	0.133 ()	0.306 ()	1.187	1.520 ()	0.072 ()	0.561 ()
Caribbean Reef	-0.164	-0.778 ()	0.245 ()	1.095	-0.687 ()	0.297 ()	-3.140 (*)
St. Martin Island	-0.135	0.723 ()	0.226 ()	1.181	-3.000 ()	0.005 (*)	-5.488 (*)
UK grassland	-0.182	1.093 ()	0.142 ()	1.266	-1.412 ()	0.069 ()	-0.985 ()
Chesapeake bay	-0.115	-0.510 ()	0.299 ()	1.080	1.015 ()	0.162 ()	1.191 ()
Northwest Shelf	-0.082	-5.125 (*)	0.000 (*)	1.024	5.000 (*)	0.001 (*)	-0.495 ()
Coachella Valley	0.050	-4.548 (*)	0.000 (*)	0.990	4.272 (*)	0.000 (*)	0.031 ()
St. Marks Forest	0.116	-3.379 (*)	0.000 (*)	0.984	2.733 ()	0.002 (*)	-0.205 ()
Biological - other (4)							
Macaque cortex [1]	-0.295	-3.167 (*)	0.001 (*)	1.664	0.076 ()	0.484 ()	-2.909 ()
C.elegans metabolic network [2]	-0.220	4.434 (*)	0.001 (*)	1.736	-1.500 ()	0.166 ()	0.5 ()
C.elegans neuronal connectivity [3]	-0.163	7.443 (*)	0.000 (*)	1.908	-12.446 (*)	0.000 (*)	-3.875 (*)
Yeast metabolic [4]	-0.076	-	-	1.411	-	-	-
Social (12)							
Facebook friendship [5]	-0.712	-	-	4.077	-	-	-
Karate club [6]	-0.476	3.744 (*)	0.000 (*)	1.568	-3.487 (*)	0.000 (*)	-0.303 ()
Kaitiaki web relations [7]	-0.245	1.698 (*)	0.042 (*)	0.203	-0.904 ()	0.165 ()	0.287 ()
Political blogs [8]	-0.221	-	-	1.549	-	-	
Ownership network [9]	-0.181	-	-	2.315	-	-	
Consulting firm [10]	-0.167	-	-	1.073	-	-	-
Political books in th US [8]	-0.128	-0.622 ()	0.229 ()	1.103	0.800 ()	0.189 ()	0.919 ()
Dolphins relationships [11]	-0.044	0.149 ()	0.460 ()	1.164	-1.020 ()	0.157 ()	-1.480 ()
Jazz musicians [12]	0.020	-7.000 (*)	0.000 (*)	0.997	4.631 (*)	0.000 (*)	1.631 ()

Football games [13]	0.162	-4.526 (*)	0.000 (*)	0.999	2.000 ()	0.002 (*)	-3.832 (*)
Net-science arXiv [14]	0.462	-	-	0.653	-	-	-
Technical (3)							
Airports network [15]	-0.221	-	-	2.352	-	-	-
Power grid [16]	0.003	-	-	1.198	-	-	-
World flights [17]	0.051	-	-	1.134	-	-	-

Table S 1. Pearson's correlation coefficient and nestedness η for different empirical (unimodal) networks from different backgrounds appearing in Fig.4 . The z-score and P-values are calculated with respect to the random null model, and the "z-score with correlations" with the null model constraint to keep to the actual value of the Pearson's coefficient r. The asterisks $*$ stand for z-values larger than 3 (in absolute value) and P-values smaller than the threshold 0.05.

Empirical bipartite networks on Fig 4

Network	r	r z-score	r P-value	η	η z-score	η P-value	η z-score with correlations
Plant - Pollinator (28)							
Andean scrub (1) (Chile) [18]	-0.239	2.448 ()	0.009 (*)	1.409	-2.857 ()	0.005 (*)	-1.067 ()
Andean scrub (2) (Chile) [18]	-0.363	2.840 ()	0.003 (*)	1.208	-3.246 (*)	0.001 (*)	-1.595 ()
Montane forest and grassland (USA) [19]	-0.131	-2.364 ()	0.011 (*)	1.218	1.067 ()	0.131 ()	-0.442 ()
High-altitude desert (Canary Islands) [20]	-0.018	0.625 ()	0.253 ()	0.989	-0.340 ()	0.334 ()	0.397 ()
Arctic community (Canada) [21]	-0.128	1.467 ()	0.079 ()	1.246	-1.164 ()	0.116 ()	0.195 ()
Multiple Communities (Galápagos Islands) [22]	-0.638	5.444 (*)	0.000 (*)	2.416	-5.033 (*)	0.003 (*)	-1.957 ()
Xeric scrub (Argentina) [23]	-0.241	4.086 (*)	0.000 (*)	1.447	-4.104 (*)	0.000 (*)	-0.919 ()
Woody riverine veg. and xeric scrub (Argentina) [23]	-0.124	0.722 ()	0.224 ()	1.268	-1.550 ()	0.071 ()	-1.745 ()
Palm swamp community (Venezuela) [24]	-0.074	0.018 ()	0.492 ()	0.872	0.370 ()	0.382 ()	1.033 ()
Boreal forest (Canada) [25]	-0.069	1.815 ()	0.030 (*)	1.309	-0.926 ()	0.138 ()	-0.151 ()
Alpine subarctic community (sweden) [26]	-0.034	0.500 ()	0.298 ()	1.061	-0.500 ()	0.282 ()	-0.625 ()
High Arctic (Canada) [27]	-0.075	0.875 ()	0.171 ()	1.322	-2.866 ()	0.030 (*)	-2.977 ()
Medow (U.K) [28]	-0.104	0.049 ()	0.434 ()	1.442	-1.049 ()	0.156 ()	-0.658 ()
Arctic community (Canada) [29]	-0.104	-1.720 ()	0.031 (*)	0.821	1.472 ()	0.069 ()	-0.485 ()
Deciduous forest (USA) [30]	-0.040	-0.571 ()	0.307 ()	1.130	0.544 ()	0.297 ()	0.130 ()
Coastal forest (Mauritius Island) [31]	-0.488	2.278 ()	0.020 (*)	1.257	-3.378 (*)	0.002 (*)	-2.143 ()
Upland grassland (South Africa) [32]	-0.041	-1.000 ()	0.180 ()	1.180	0.885 ()	0.232 ()	0.017 ()
Maple-oak woodland (USA) [33]	-0.037	0.500 ()	0.320 ()	1.114	-0.166 ()	0.426 ()	0.578 ()
Snowy Mountains of Australia [34]	-0.139	0.778 ()	0.236 ()	1.165	-1.432 ()	0.076 ()	-0.804 ()
Peat bog (Canada) [35]	0.003	-2.857 ()	0.002 (*)	0.905	1.722 ()	0.035 (*)	-0.367 ()
Arroyo Goye (Argentina) [36–38]	-0.034	-0.091 ()	0.497 ()	0.114	-0.125 ()	0.446 ()	-0.507 ()
Cerro Lopez (Argentina) [36–38]	-0.030	0.071 ()	0.412 ()	0.133	-0.250 ()	0.352 ()	-0.078 ()
Llao Llao (Argentina) [36–38]	-0.059	0.182 ()	0.375 ()	0.124	-0.375 ()	0.317 ()	-0.504 ()
Mascardi (c) (Argentina) [36–38]	-0.034	0.500 ()	0.307 ()	0.083	0.000 ()	0.417 ()	1.300 ()
Mascardi (nc) (Argentina) [36–38]	-0.023	-1.400 ()	0.045 (*)	0.151	1.555 ()	0.040 (*)	0.937 ()
Quetrihue (c) (Argentina) [36–38]	-0.051	0.900 ()	0.144 ()	0.104	-1.428 ()	0.081 ()	-0.806 ()
Quetrihue (nc) (Argentina) [36–38]	-0.043	1.636 ()	0.073 ()	0.079	-1.750 ()	0.053 ()	-0.627 ()
Safariland (Argentina) [36–38]	-0.035	0.031 ()	0.432 ()	0.097	-0.142 ()	0.406 ()	-0.182 ()
Plant - Hervibore (4)							

Arid grasslands in Marathon (USA) [39]	-0.168	4.571 (*)	0.000 (*)	1.193	-1.256 ()	0.112 ()	2.696 ()
Arid grasslands in Altuda (USA) [39]	-0.215	3.200 (*)	0.001 (*)	1.316	-1.489 ()	0.048 (*)	1.226 ()
Whole country (Britain) [40]	-0.016	-0.500 ()	0.198 ()	1.100	0.875 ()	0.177 ()	0.693 ()
Whole country (Finland) [40]	-0.055	0.651 ()	0.275 ()	1.420	0.000 ()	0.240 ()	-0.320 ()
Plant - Seed Disperser (5)							
Forest (Papa New Guinea) [41]	-0.030	0.075 ()	0.427 ()	1.131	-0.588 ()	0.246 ()	-0.537 ()
Semideciduous tropical forest (Panama) [42]	-0.689	0.276 ()	0.322 ()	1.081	-0.14 ()	0.449 ()	-0.950 ()
Neotropical forest (Trinidad) [43]	-0.026	0.858 ()	0.192 ()	1.077	-0.736 ()	0.231 ()	-0.178 ()
Birds and Berries. Calton (England) [43]	-0.087	0.500 ()	0.346 ()	0.796	0.333 ()	0.358 ()	0.965 ()
Temperate woodland (Britain) [44]	-0.459	0.814 ()	0.266 ()	0.926	-0.561 ()	0.470 ()	1.559 ()
Anemone-Fish (1)							
Coral reefs [45]	-0.064	0.063 ()	0.483 ()	1.085	-0.633 ()	0.322 ()	-1.025 ()

Table S 2. Pearson's correlation and nestedness η for empirical bimodal networks from different backgrounds appearing in Fig.4 . The z-score and P-values are calculated with respect to the random null model, and the "z-score with correlations" with the null model restricted to the actual correlation coefficient r of the network. As above, the asterisks $*$ stand for z-values larger than 3 (in absolute value) and P-values smaller than the threshold 0.05.

References

1. Harriger L, van den Heuvel MP, Sporns O (2012) Rich club organization of macaque cerebral cortex and its role in network communication. Plos One 7: e46497.

2. Duch J, Arenas A (2005) Community identification using extremal optimization. Physical Review E 72: 027104.

3. Watts DJ, Strogatz SH (1998) Collective dynamics of 'small-world' networks. Nature 393: 440-442.

4. Jeong H, Mason S, Barabási A, Oltvai ZN (2001) Centrality and lethality of protein networks. Nature 411: 41.

5. McAuley J, Leskovec J (2012) Learning to discover social circles in ego networks. In: Advances in Neural Information Processing Systems. pp. 548–556.

6. Zachary WW (1977) An information flow model for conflict and fission in small groups. Journal of Anthropological Research 33: 452-473.

7. Kaitiaki trust network. available at http://wwwtrustletorg/datasets/kaitiaki/ Accesed 2013 Jul 31 .

8. Adamic LA, Glance N (2005) The political blogosphere and the 2004 us election. Proceedings of the WWW-2005 Workshop on the Weblogging Ecosystem .

9. Norlen K, Lucas G, Gebbie M, Chuang J (2002) Eva: Extraction, visualization and analysis of the telecommunications and media ownership network. Proceedings of International Telecommunications Society 14th Biennial Conference, Seoul Korea .

10. Cross R, Parker A (2004) The Hidden Power of Social Networks. Boston: Harvard Business School Press.

11. Lusseau D, Schneider K, Boisseau OJ, Haase P, Slooten E, et al. (2003) The bottlenose dolphin community of doubtful sound features a large proportion of long-lasting associations. Behavioral Ecology and Sociobiology 54: 396-405.

12. PGleiser, Danon L (2003) Community structure in jazz. Adv Complex Syst 6: 565.

13. Girvan M, Newman MEJ (2002) Community structure in social and biological networks. Proc Natl Acad Sci USA 99: 7821-1826.

14. Newman MEJ (2006) Finding community structure in networks using the eigenvectors of matrices. Phys Rev E 74: 036104.

15. Opsahl T (2011) Why anchorage is not (that) important: Binary ties and sample selection. Available at http://wpme/poFcY-Vw Accesed 2013 Aug 10 .

16. Watts DJ, Strogatz SH (1998) Collective dynamics of 'small-world' networks. Nature 393: 440-442.

17. Opsahl T, Agneessens F, Skvoretz J (2010) Node centrality in weighted networks: Generalizing degree and shortest paths. Social Networks 3: 245–251.

18. Arroyo MTK, Primack RB, , Armesto JJ (1982) Community studies in pollination ecology in the high temperate andes of central chile. i. pollination mechanisms and altitudinal variation. American Journal of Botany 69: 82-97.

19. Clements RE, Long FL (1923) Experimental pollination. An outline of the ecology of flowers and insects. Washington D.C., USA: Carnegie Institute of Washington.

20. Dupont Y, Hansen D, Olesen J (2003) Structure of a plant-flower-visitor network in the high-altitude sub-alpine desert of tenerife, canary islands. Ecography 26: 301-310.

21. Hocking B (1968) Insect-flower associations in the high arctic with special reference to nectar. Oikos 19: 359-388.

22. McCullen CK (1993) Flower-visiting insects of the galapagos islands. Pan-Pacific Entomologist 69: 95-106.

23. Medan D, Montaldo NH, Devoto M, Mantese A, Vasellati V, et al. (2002) Plant-pollinator relationships at two altitudes in the andes of mendoza, argentina. Arctic Antarctic and Alpine Research 34: 233-24.

24. Ramirez N, Brito Y (1992) Pollination biology in a palm swamp community in the venezuelan central plains. Botanical Journal of the Linnean Society 110: 277-302.

25. Barrett SCH, Helenurm K (1987) The reproductive-biology of boreal forest herbs.1. breeding systems and pollination. Canadian Journal of Botany 65: 2036-2046.

26. Elberling H, Olesen JM (1999) The structure of a high latitude plant-flower visitor system: the dominance of flies. Ecography 22: 314-323.

27. Kevan PG (1970) High arctic insect-flower visitor relations: the inter-relationships of arthropods and flowers at Lake Hazen, Ellesmere Island, Northwest Territories, Canada. Ph.D. thesis thesis, University of Alberta.

28. Memmott J (1999) The structure of a plant-pollinator food web. Ecology Letters 2: 276-280.

29. Mosquin T, Martin JEH (1967) Observations on the pollination biology of plants on melville island, n.w.t., canada. Canadian Field Naturalist 81: 201-205.

30. Motten AF (1982) Pollination Ecology of the Spring Wildflower Community in the Deciduous Forests of Piedmont North Carolina. North Carolina, USA: Doctoral Dissertation thesis, Duke University, Duhram.

31. Olesen JM, Eskildsen LI, Venkatasamy S (2002) Invasion of pollination networks on oceanic islands: importance of invader complexes and endemic super generalists. Diversity and Distributions 8: 181-192.

32. Ollerton J, Johnson SD, Cranmer L, Kellie S (2003) The pollination ecology of an assemblage of grassland asclepiads in south africa. Annals of Botany 92: 807-834.

33. Schemske DW, Willson MF, Melampy MN, Miller LJ, Verner L, et al. (1978) Flowering ecology of some spring woodland herbs. Ecology 59: 351-366.

34. Inouye DW, Pyke GH (1988) Pollination biology in the snowy mountains of australia: comparisons with montane colorado, usa. Australian Journal of Ecology 13.

35. Small E (1976) Insect pollinators of the mer bleue peat bog of ottawa. Canadian Field Naturalist 90: 22-28.

36. Vázquez DP (2002) Interactions among Introduced Ungulates, Plants, and Pollinators: A Field Study in the Temperate Forest of the Southern Andes. Doctoral Dissertation thesis. Knoxville, Tennessee, USA: University of Tennessee, USA.

37. Vázquez DP, Simberloff D (2002) Ecological specialization and susceptibility to disturbance: conjectures and refutations. American Naturalist 159: 606-623.

38. Vázquez DP, Simberloff D (2003) Changes in interaction biodiversity induced by an introduced ungulate. Ecology Letters 6: 1077-1083.

39. Joern A (1979) Feeding patterns in grasshoppers (orthoptera: Acrididae): factors influencing diet specialization. Oecologia 38: 325-347.

40. Leather S (1991) Feeding specialisation and host distribution of british and finnish prunus feeding macrolepidoptera. OIKOS 60: 40-48.

41. Beehler B (1983) Frugivory and polygamy in birds of paradise. The Auk 100: 1-12.

42. Poulin B, Wright SJ, Lefebvre G, Calderon O (1999) Interspecific synchrony and asynchrony in the fruiting phenologies of congeneric bird-dispersed plants in panama. Journal of Tropical Ecology 15: 213-227.

43. Snow BK, Snow DW (1971) The feeding ecology of tanagers and honeycreeperes in trinidad. The Auk 88: 291-322.

44. Sorensen A (1981) Interactions between birds and fruit in a temperate woodland. Oecologia 50: 242-249.

45. Ollerton J, McCollin D, Fautin D, Allen G (2006) Finding nemo: nestedness engendered by mutualistic organisation in anemonefish and their hosts. Proceedings of the Royal Society B : doi:10.1098.
